# The development of simple anthropometric measures to diagnose antiretroviral therapy-associated lipodystrophy in resource limited settings

**DOI:** 10.1186/1742-6405-11-26

**Published:** 2014-08-04

**Authors:** Zulfa Abrahams, Joel A Dave, Gary Maartens, Maia Lesosky, Naomi S Levitt

**Affiliations:** 1 Division of Diabetic Medicine and Endocrinology, Department of Medicine, University of Cape Town, Cape Town, South Africa; 2 Division of Clinical Pharmacology, Department of Medicine, University of Cape Town, Cape Town, South Africa; 3 Department of Medicine, University of Cape Town, Cape Town, South Africa

**Keywords:** HIV, Lipoatrophy, Lipohypertrophy, Lipodystrophy, DXA, Anthropometry, Sub-Saharan africa, Antiretroviral therapy

## Abstract

**Background:**

Lipohypertrophy does not appear to be an adverse ART reaction while lipoatrophy is clearly associated with the use of stavudine (d4T) and zidovudine (AZT). In low and middle income countries d4T has only recently been phased out and AZT is still widely being used. Several case definitions have been developed to diagnose lipodystrophy, but none of them are generalizable to sub-Saharan Africa where black women have less visceral adipose tissue and more subcutaneous adipose tissue than white women. We aimed to develop a simple, objective measure to define lipoatrophy and lipohypertrophy by comparing patient report to anthropometric and dual-energy X-ray absorptiometry (DXA) -derived variables.

**Methods:**

DXA and anthropometric measures were obtained in a cross sectional sample of black HIV-infected South African men (n = 116) and women (n = 434) on ART. Self-reported information on fat gain or fat loss was collected using a standard questionnaire. Receiver operating characteristic (ROC) curves were used to describe the performance of anthropometric and DXA-derived variables using patient reported lipoatrophy and lipohypertrophy as the reference standard.

**Results:**

Lipoatrophy and lipohypertrophy were more common in women (25% and 33% respectively) than in men (10% and 13% respectively). There were insufficient numbers of men with DXA scans for meaningful analysis. The best predictors of lipoatrophy in women were the anthropometric variables tricep (AUC = 0.725) and thigh skinfold (AUC =0.720) thicknesses; and the DXA-derived variables percentage lower limb fat (AUC = 0.705) and percentage lower limb fat/height (AUC = 0.713). The best predictors of lipohypertrophy in women were the anthropometric variable waist/hip ratio (AUC = 0.645) and the DXA-derived variable percentage trunk fat/percentage limb fat (AUC = 0.647).

**Conclusions:**

We were able to develop simple, anthropometric measures for defining lipoatrophy and lipohypertrophy, using a sample of black HIV-infected South African women with DXA scans. This is of particular relevance in resource limited settings, where health professionals need simple and inexpensive methods of diagnosing patients with lipoatrophy and lipohypertrophy.

## Background

Antiretroviral therapy (ART) has significantly reduced the morbidity and mortality of people infected with HIV [1], however, long-term use of ART has been associated with a number of metabolic complications such as dysglycaemia, insulin resistance, dyslipidaemia and lipodystrophy [2]. Lipodystrophy is characterized by either subcutaneous fat loss (lipoatrophy), which is most noticeable in the face, limbs, and buttocks, or fat accumulation (lipohypertrophy) seen in the abdomen, breast or posterior neck, or a combination of both [3,4].

Both subjective and objective methods have been used to diagnose lipodystrophy, resulting in a number of case definitions. The most widely used subjective methods of diagnosis are patient perception and report [5,6], physician examination and report [7], and physician confirmation of patient report [8-11]. Objective measures include imaging by dual-energy X-ray absorptiometry (DXA) [6,12,13] and computed tomography (CT) scans [12,14]. These imaging measures are expensive and not widely available in resource limited settings. Anthropometric and DXA-derived variables have also been developed, in an attempt to provide standard measures of defining lipodystrophy [15-17]. Furthermore, criteria established to define lipodystrophy did not include data from any African country. These diagnostic criteria may not be generalizable to sub-Saharan Africans, as there are important ethnic differences in fat distribution, especially in black women who have less visceral adipose tissue and more subcutaneous adipose tissue than white women [18-20].

Lipohypertrophy does not appear to be an adverse ART reaction as participants on different ART drug regimens gained similar amounts of trunk fat over time [21]. Lipoatrophy, in contrast is clearly an adverse ART reaction. The use of stavudine (d4T) and zidovudine (AZT) is associated with subcutaneous fat loss and is partially reversed after changing to abacavir or tenofovir [21,22]. Lipoatrophy remains common in low and middle income countries where d4T has only recently been phased out and AZT is still widely used [23]. However, even if lipohypertrophy is not associated with ART, lipodystrophy, and lipoatrophy in particular, is independently associated with an increased risk of vascular disease [24,25]. Therefore recognising lipodystrophy is important to identify patients at risk for vascular disease so that screening can be targeted for other vascular risk factors, while recognising lipoatrophy is important so that d4T or AZT can be substituted.

The aim of our study was to develop a simple, objective measure to define lipoatrophy and lipohypertrophy by comparing patient report to anthropometric and DXA-derived variables in a sample of black South Africans on ART.

## Results

Participant characteristics are presented in Table 1. The study sample consisted of 550 participants on ART. Based on patient report, 121 (22%) had lipoatrophy and 157 (29%) had lipohypertrophy. Both lipoatrophy and lipohypertrophy were significantly more common in females than in males (p ≤ 0.001). Participants with lipoatrophy had spent a significantly longer period of time on ART (25 vs. 17 months) and a longer time on d4T (15.5 vs. 13 months).

**Table 1:** Characteristics of participants on ART

**Variable**	Lipoatrophy*		P-value**	Lipohypertrophy***		P-value**
	**With n = 121 Median [IQR]**	**Without n = 429 Median [IQR]**		**With n = 157 Median [IQR]**	**Without n = 393 Median [IQR]**	
Age	34 [30–42]	35 [30–41]	0.256	34 [29–41]	35 [30–41]	0.198
Current CD4 count	397 [249–539]	315 [218–481]	0.023	389 [248–548]	314 [220–481]	0.015
Time on ART (months)	25 [14–32]	17 [10–27]	0.001	20 [12–31]	17 [11–28]	0.080
Time on Stavudine (months)	15.5 [10–26]	13 [8–19]	0.004	13 [8–21]	13 [9–20]	0.961
	**n [%]**	**n [%]**		**n [%]**	**n [%]**	
Sex						
Males	12 [10.34]	104 [89.66]	0.001	15 [12.93]	101 [87.07]	<0.001
Females	109 [25.12]	325 [74.88]		142 [32.72]	292 [67.28]	
Highest standard passed						
No schooling	6 [18.8]	26 [81.3]	0.289	9 [28.13]	23 [71.88]	0.272
Primary school	14 [15.4]	77 [84.6]		20 [21.98]	71 [78.02]	
Secondary school	96 [23.4]	315 [76.6]		121 [29.44]	290 [70.56]	
Tertiary	5 [31.3]	11 [68.8]		7 [43.75]	9 [56.25]	
ART Regimen						
First-line	99 [22.30]	345 [77.70]	0.730	130 [29.28]	314 [70.72]	0.608
Second-line	22 [20.75]	84 [79.25]		27 [25.47]	79 [74.53]	

*based on patient report, those with moderate or severe fat loss in 2 or more regions.

**Wilcoxon Rank-sum (Mann–Whitney) tests for continuous variables and chi-square tests for binary variables.

***based on patient report, those with moderate or severe fat gain in 2 or more regions.

Anthropometric variables are shown separately for women and men (Tables 2 and 3 respectively). In women, all median skinfold measurements, with the exception of sub-scapular skinfold thickness, were significantly lower in participants with lipoatrophy compared with those without lipoatrophy. Measurements for waist circumference, waist/hip ratio and supra-iliac skinfold thickness were significantly higher in women with lipohypertrophy compared with those without lipohypertrophy. There were no statistically significant differences in anthropometric variables in males with and without lipoatrophy (Table 3). Males with lipohypertrophy had a significantly (P = 0.008) greater thigh circumference than those without (13.5 mm vs. 8.1 mm).

**Table 2:** Anthropometric measurements of female participants on ART

**Variable**	Lipoatrophy*		P-value**	Lipohypertrophy***		P-value**
	**With n = 106 Median [IQR]**	**Without n = 312 Median [IQR]**		**With n = 142 Median [IQR]**	**Without n = 292 Median [IQR]**	
Height (m)	1.6 [1.5-1.6]	1.6 [1.5-1.6]	0.603	1.6 [1.6-1.6]	1.6 [1.5-1.6]	0.112
Weight (kg)	65.4 [56.0-74.5]	68.1 [58.9-80.9]	0.019	69.5 [60.7-80.4]	67.1 [57.6-79.3]	0.127
BMI	26.2 [23.8-29.3]	27.1 [24.1-31.6]	0.036	27.4 [24.6-31.3]	26.65 [23.9-31.2]	0.187
Sagittal Abdominal Diameter (cm)	20.5 [19.0-23.0]	20.0 [18.5-23.0]	0.552	21 [19–24]	20 [18–23]	0.004
**Circumferences**						
Waist (cm)	86.3 [79.5-96.3]	87.0 [78.5-97.0]	0.704	90.7 [80.3-98.5]	86 [78.4-95]	0.003
Hip (cm)	98.0 [92.0-104.0]	102.0 [95.0-112.0]	<0.001	100 [95–110]	101 [94–110]	0.824
Waist/hip ratio	0.90 [0.83-0.94]	0.85 [0.80-0.90]	<0.001	0.89 [0.83-0.93]	0.84 [0.79-0.90]	<0.001
Mid-upper arm (cm)	27.0 [25.0-29.0]	29.0 [26.5-32.0]	<0.001	28 [26–32]	28 [26–32]	0.789
Mid-thigh (cm)	54.5 [51.0-59.0]	57.0 [53.0-63.0]	<0.001	56 [52–61]	57[ 52–63]	0.589
**Skinfolds**						
Bicep (mm)	6.0 [4.4-8.4]	8.1 [5.7-11.7]	<0.001	7.1 [5.2-10.7]	8 [5.4-11.45]	0.189
Tricep (mm)	13.3 [9.5-17.4]	18.4 [13.6-26.0]	<0.001	16.2 [12–22]	18.3 [13–25.15]	0.059
Abdomen (mm)	20.4 [11.9-30.0]	24.4 [16.8-35.3]	0.001	24.1 [15.9-35.8]	23.6 [15.0-31.2]	0.210
Thigh (mm)	23.8 [15.4-32.8]	34.7 [24.2-44.7]	<0.001	29.8 [20.5-42.5]	32.0 [22.5-43.5]	0.359
Sub-Scapular (mm)	16.9 [12.5-23.0]	19.2 [12.2-28.8]	0.058	20 [14.8-29.4]	18.5 [11.7-27.1]	0.050
Supra-iliac (mm)	13.2 [7.7-18.7]	15.1 [8.8-22.8]	0.033	16.6 [9.9-24.0]	13.9 [8.2-21.7]	0.022
Calf (mm)	10.8 [8.0-17.5]	17.5 [12.2-24.2]	<0.001	15.6 [9.2-21.3]	17.3 [11.4-23.6]	0.061

*based on patient report, those with moderate or severe fat loss in 2 or more regions.

**Wilcoxon Rank-sum (Mann–Whitney) tests for continuous variables and chi-square tests for binary variables.

***based on patient report, those with moderate or severe fat gain in 2 or more regions.

**Table 3:** Anthropometric measurements of male participants on ART

**Variable**	Lipoatrophy*		P-value**	Lipohypertrophy***		P-value**
	**With n = 12 Median [IQR]**	**Without n = 104 Median [IQR]**		**With n = 15 Median [IQR]**	**Without n = 101 Median [IQR]**	
Height (m)	1.7 [1.6-1.7]	1.7 [1.6-1.7]	0.942	1.7 [1.6-1.8]	1.7 [1.7-1.7]	0.954
Weight (kg)	65.5 [59.7-68.6]	63.5 [57.0-74.4]	0.895	67.4 [57.8-78.3]	62.7 [57.3-73.4]	0.408
BMI	23.3 [20.2-23.9]	22.4 [20.6-25.2]	0.772	23.6 [21.1-25.5]	22.3 [20.5-24.8]	0.324
Sagittal Abdominal Diameter (cm)	17.0 [16.0-19.5]	18.0 [17.0-20.0]	0.384	17.0 [16.0-22.0]	18.0 [17.0-20.0]	0.967
**Circumferences**						
Waist (cm)	81.75 [77–84.9]	80.4 [75.0-90.2]	0.953	83.8 [75.0-97.0]	79.8 [75.8-89.5]	0.338
Hip (cm)	90 [87–93.5]	90.0 [85.0-97.0]	0.768	91.0 [85.0-97.0]	90.0 [85.0-96.0]	0.556
Waist/hip ratio	0.89 [0.85-0.95]	0.91 [0.87-0.94]	0.740	0.93 [0.86-0.97]	0.90 [0.86-0.94]	0.325
Mid-upper arm (cm)	27.0 [24–28.5]	26.0 [24.0-29.0]	0.877	27.0 [25.0-29.0]	26.0 [24.0-29.0]	0.817
Mid-thigh (cm)	50.5 [43.5-52.5]	49.0 [46.0-54.0]	0.401	49.0 [46.0-56.0]	49.0 [46.0-54.0]	0.567
**Skinfolds**						
Bicep (mm)	3.9 [3.4-4.2]	3.5 [3.0-4.5]	0.329	4.1 [3.2-4.8]	3.5 [3.0-4.3]	0.180
Tricep (mm)	6.8 [5.6-8.2]	6.4 [5.1-9.3]	0.921	8.4 [5.0-11.4]	6.4 [5.1-8.7]	0.444
Abdomen (mm)	13.5 [10.5-16.9]	12.3 [8.5-30.1]	1.00	15.8 [9.3-28.2]	12.3 [8.5 -18.1]	0.309
Thigh (mm)	8.4 [7.2-14.8]	8.5 [6.0-12.4]	0.490	13.5 [8.4-21.1]	8.1 [6.0-11.3]	0.008
Sub-Scapular (mm)	9.4 [6.5-13.2]	8.3 [6.3-13.4]	0.605	10.5 [5.7-18.0]	8.2 [6.5-11.1]	0.770
Supra-iliac (mm)	6.3 [4.4-6.8]	6.2 [4.9-9.4]	0.196	6.4 [4.7-12.1]	6.2 [4.9-8.5]	0.934
Calf (mm)	5.5 [4.8-9.0]	5.8 [4.4-8.2]	0.739	6.8 [5.4-10.5]	5.5 [4.4-7.7]	0.094

*based on patient report, those with moderate or severe fat loss in 2 or more regions.

**Wilcoxon Rank-sum (Mann–Whitney) tests for continuous variables and chi-square tests for binary variables.

***based on patient report, those with moderate or severe fat gain in 2 or more regions.

**Figure 1:**
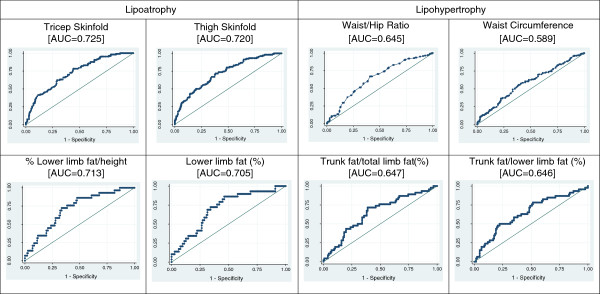
ROC curves for the 2 anthropometric and DXA-derived variables with the highest AUC for lipoatrophy and lipohypertrophy in female participants on ART.

**Figure 2:**
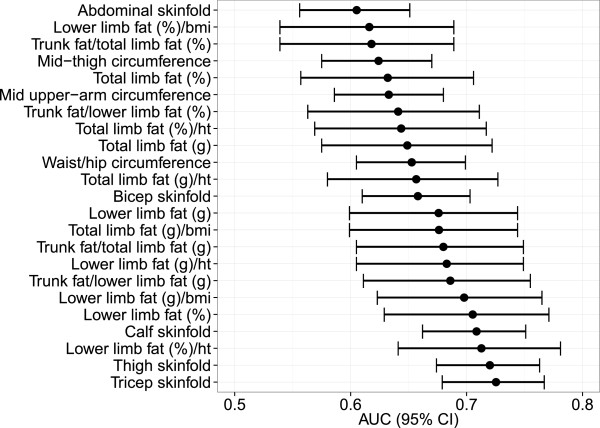
Lipoatrophy variables for female participants on ART with ROC AUCs of ≥0.6 and their 95% confidence intervals in descending order of AUC.

DXA-derived measures are shown for women only (Table 4), as there were insufficient numbers of men with DXA scans for meaningful analysis. Women with lipoatrophy as well as those with lipohypertrophy, had significantly higher percentage trunk fat/lower limb fat and percentage trunk fat/total limb fat and significantly lower percentage lower limb fat/BMI. Women with lipoatrophy had significantly less percentage limb fat while women with lipohypertrophy had significantly more percentage trunk fat.ROC curves for lipoatrophy and lipohypertrophy were generated and reported in female participants for anthropometric and DXA-derived variables with the highest AUC’s (Figure 1). For lipoatrophy, the two anthropometric variables with the highest AUC were tricep skinfold thickness (AUC = 0.725) and thigh skinfold thickness (AUC = 0.720) and for lipohypertrophy they were waist/hip ratio (AUC = 0.645) and waist circumference (AUC = 0.589). For lipoatrophy, the two DXA-derived variables with the highest AUC were the percentage of lower limb fat standardised to height (AUC = 0.713) and percentage lower limb fat (AUC = 0.705) and for lipohypertrophy they were percentage trunk fat/percentage total limb fat (AUC = 0.647) and percentage trunk fat/ percentage lower limb fat (AUC = 0.646). An illustration of anthropometric and DXA-derived variables in females is shown in Figure 2.

**Table 4:** DXA-derived measurements of female participants on ART

**Variable**	Lipoatrophy*		P-value**	Lipohypertrophy***		P-value**
	**With n = 29 Median [IQR]**	**Without n = 143 Median [IQR]**		**With n = 46 Median [IQR]**	**Without n = 126 Median [IQR]**	
**DXA-derived measures**						
Arm fat (%)	37.4 [30.8-44.1]	38.3 [31.8-47.1]	0.380	39.8 [31.5-48.3]	38.1 [31.7-45.1]	0.319
Lower limb fat (%)	37.6 [31.5-40.6]	43.4 [36.6-49.7]	0.001	39.2 [33.3-49.7]	41.8 [36.3-48.8]	0.761
Trunk fat (%)	34.7 [29.9-38.0]	35.1 [27.9-43.1]	0.719	37.9 [30.7-45.3]	34.4 [26.6-40.4]	0.019
Lower limb fat/ht (%)	23.6 [19.7-25.4]	27.4 [23.4-31.5]	0.001	25.4 [21.9-31.1]	26.5 [23.2-29.7]	0.631
Total limb fat/ht (%)	22.7 [20.8-27.0]	25.4 [22.0-29.9]	0.015	25.4 [20.8-31.1]	25.2 [21.8-29.1]	0.825
Lower limb fat/BMI (%)	1.3 [1.2-1.7]	1.6 [1.3-1.8]	0.049	1.4 [1.2-1.6]	1.6 [1.3-1.8]	0.019
Total limb fat/BMI (%)	1.3 [1.3-1.6]	1.5 [1.3-1.6]	0.193	1.3 [1.2-1.5]	1.5 [1.3-1.7]	0.006
Trunk fat/lower limb fat (%)	0.94 [0.76-1.15]	0.83 [0.66-0.96]	0.017	0.93 [0.80-1.10]	0.81 [0.66-0.94]	0.003
Trunk fat/ total limb fat (%)	1.0 [0.8-1.0]	0.9 [0.7-1.0]	0.045	0.93 [0.85-1.03]	0.85 [0.72-0.97]	0.003

*based on patient report, those with moderate or severe fat loss in 2 or more regions.

**Wilcoxon Rank-sum (Mann–Whitney) tests for continuous variables and chi-square tests for binary variables.

***based on patient report, those with moderate or severe fat gain in 2 or more regions.

Optimum cut-points for lipoatrophy and lipohypertrophy variables, based on likelihood ratios, were selected. Table 5 shows the sensitivity, specificity, likelihood ratios and predictive values for the two anthropometric and DXA-derived variables with the highest AUC for lipoatrophy and lipohypertrophy at the optimum cut-points.

**Table 5:** Variables for prediction and classification used to identify lipoatrophy and lipohypertrophy cut-points

**Lipoatrophy variable cut-point**	**N**	**Sensitivity**	**Specificity**	**Likelihood ratio positive test**	**Likelihood ratio negative test**	**Positive predictive value**	**Negative predictive value**	**% correctly classified**
Tricep skinfold ≤14.5 mm	418	0.62	0.71	2.13	0.53	0.42	0.85	68.66*
Thigh skinfold ≤28.0 mm	417	0.67	0.65	1.93	0.51	0.39	0.85	65.70*
% Lower limb fat/height ≤ 24.7	418	0.19	0.85	1.28	0.95	0.30	0.76	68.42*
Lower limb fat (%) ≤ 39.1	418	0.19	0.84	1.20	0.96	0.29	0.75	67.70*
**Lipohypertrophy variable cut-point**								
Waist/Hip Ratio ≥ 0.899	434	0.44	0.75	1.75	0.75	0.46	0.73	64.75**
Waist Circumference ≥ 88.5 cm	434	0.57	0.59	1.40	0.73	0.41	0.73	58.53**
Trunk fat/total limb fat (%) ≥0.8895	172	0.50	0.70	1.66	0.72	0.38	0.79	64.5**
Trunk fat/leg fat (%) ≥ 0.8816	172	0.72	0.61	1.84	0.46	0.40	0.86	64.0**

*percentage of those classified with and without lipoatrophy using the new cut-point compared to those defined by subject-report.

**percentage of those classified with and without lipohypertrophy using the new cut-point compared to those defined by subject-report.

## Discussion

We showed that simple anthropometric measures were at least as good as DXA-derived measures to diagnose lipoatrophy and lipohypertrophy in African women on ART. The best predictors of lipoatrophy in women were the anthropometric variables tricep and thigh skinfold thicknesses; and the DXA-derived variables percentage lower limb fat and percentage lower limb fat/height. The best predictors of lipohypertrophy in women were the anthropometric variable waist/hip ratio and the DXA-derived variable percentage trunk fat/percentage limb fat. Women with lipoatrophy had considerably smaller limb circumferences, limb skinfold thicknesses and lower percentages of limb fat than women without lipoatrophy, despite similar BMIs. Lipoatrophy and lipohypertrophy were both more common in women than in the small sample of men.

Previous studies, conducted in high-income countries, developed objective measures for lipodystrophy, thus combining lipoatrophic and lipohypertrophic individuals [15-17]. They proposed the use of fat mass ratio (FMR), defined as the ratio between the percentage of trunk fat mass and the percentage of lower-limb fat mass. We however sought to investigate lipoatrophy and lipohypertrophy as separate entities. Identification of lipoatrophy is important as it is an adverse antiretroviral drug reaction, which improves on switching antiretroviral drugs [21]. Although lipohypertrophy is thought to be a consequence of treating HIV infection rather than an adverse antiretroviral drug reaction [21], like lipoatrophy, it is associated with an increased risk of vascular disease [26] therefore it is worth identifying so that appropriate screening and prevention interventions can be implemented.

Despite the subjective nature of assessing lipoatrophy and lipohypertrophy by using patient self-report, previous studies have shown a strong correlation between patient and physician reported lipodystrophy scores [27-29]. In South Africa, as well as in many other African countries, nurses, rather than physicians, prescribe antiretroviral therapy and follow up patients. For these reasons we used patient self-report [5,6] as the reference measure to define lipoatrophy and lipohypertrophy.

Our study, like several others [11,21], showed a significant association between lipoatrophy and time on ART, and time on d4T in particular. As South Africa has only recently phased out d4T, and AZT is still being used, it is not unexpected that a quarter of the women and a tenth of the men, had lipoatrophy. The prevalence of lipoatrophy found in this study is not easy to compare with other studies as studies from high-income countries focussed on men [12,24], while most studies from Africa looked at the prevalence of lipodystrophy [30-32] rather than studying the two entities of lipoatrophy and lipodystrophy separately. Our finding that tricep skinfold thickness was a predictor of lipoatrophy is supported by other studies. George et al. [33], using a small sample of HIV-infected South Africans, found that after 2 years of exposure to ART, patients had significantly decreased tricep skinfold thicknesses. Similarly, a Ugandan study using a sample of HIV-infected men and women [32], found that decreased tricep skinfold thicknesses was associated with the use of AZT.

There were some limitations to our study. The cross sectional design, while allowing us to make associations, does not allow us to infer causality. With changes in fat distribution, repeated objective measures would have given us a better reference standard than patient report, even though patient report is commonly used [5,6]. We did not have enough men with lipoatrophy or lipohypertrophy, to explore predictive anthropometric and DXA-derived variables. Finally, the likelihood ratios for the most predictive anthropometric and DXA-derived variables were only weakly diagnostic of self-report lipoatrophy and lipohypertrophy. Future research of longitudinal studies in African cohorts, using changes in DXA-derived variables as the reference standard, is needed to confirm the value of anthropometric measures for the diagnosis of lipoatrophy and lipohypertrophy.

## Conclusion

Using a large sample of black HIV-infected South African women who had DXA scans performed, we were able to develop anthropometric measures for defining lipoatrophy and lipohypertrophy. The development of anthropometric measures which admittedly needs training and well maintained skinfold callipers to ensure their accuracy, are of particular relevance in resource limited settings, where health professionals need simple and inexpensive methods of diagnosing patients with lipoatrophy and lipohypertrophy.

## Methods

### Participants

A convenience sample of HIV-infected black men and women presenting to ART clinics in Cape Town were selected. The recruitment procedure is described elsewhere [34]. The study sample comprised 116 male and 434 female participants on ART. At the time of the study two ART regimens were available to South Africans accessing primary health care facilities. The first-line regimen consisted of d4T, lamivudine (3TC) and efavirenz (EFV) or nevirapine, and a second-line regimen consisting of AZT with 3TC and lopinavir/ritonavir (LPV/r) [35].

### Testing procedures

Questionnaires were used to collect socio-demographic information from participants. Their clinical records at the health facilities were reviewed to obtain data on ART regimen, time on ART, and CD4 count. Self-reported information on fat gain or fat loss was collected using a standard questionnaire [8]. Lipoatrophy was defined as moderate or severe fat loss in 2 or more regions and lipohypertrophy defined as moderate or severe fat gain in two or more areas [36].

Anthropometric measurements: [weight, height, circumferences (waist, hip, mid-upper arm, and mid-thigh), skinfold thickness (bicep, tricep, subscapular, abdomen, suprailiac, thigh and calf) and sagittal abdominal diameter (SAD)] taken have previously been described [37]. DXA (Hologic Discovery-W, software version 12.7; scan region 195 × 65 cm^2^ and weight limit 160 kg) was used to measure fat mass and fat free soft tissue mass in a subsample of participants (females: n = 172; males: n = 53). DXA cut off lines positioned at anatomical markers were used to obtain fat mass for the whole body as well as for the various regions of interest. A more detailed description has been previously described [34].

### Ethical approval

The study proposal was submitted and approved by the Research Ethics Committee of the Faculty of Health Sciences at the University of Cape Town. Written informed consent was obtained from all participants prior to participation in the study.

### Data analyses

Data analysis was carried out using the STATA/SE statistical software package version 12.0 (StataCorp., College Station, TX, USA). Data were collected between February 2007 and June 2009. Participants were categorised into those with and those without lipoatrophy. Continuous variables were described as medians and inter-quartile ranges (IQR), and were compared using Wilcoxon Rank Sum tests. Binary variables were described using chi-square tests.

Receiver operating characteristic (ROC) curves were used to describe the performance of a number of anthropometric and DXA-derived variables using patient reported lipoatrophy and lipohypertrophy as the reference standard. The area under the curve (AUC) was used to assess the diagnostic performance of each variable. In addition, sensitivity, specificity, likelihood ratios and predictive values were calculated for variables with the highest AUC at the optimum cut-points. Cut-point selection was based on positive likelihood ratios.

## Competing interests

Supported by grants from the World Diabetes Foundation and the South African Department of Health. GM was supported in part by the National Research Foundation (NRF) of South Africa (grant specific unique reference number (UID) 85810). The Grant holder acknowledges that opinions, findings and conclusions or recommendations expressed in any publication generated by the NRF supported research are that of the author(s), and that the NRF accepts no liability whatsoever in this regard.

## Authors’ contributions

ZA conducted all statistical analyses, interpreted the findings and drafted the manuscript; JD, NL and GM designed and conducted the study; ML assisted with study design and data interpretation; NL, GM, JD and ML edited the manuscript and drafted revisions. All authors read and approved the manuscript.
